# Effects of housing demolition on health and medical utilization: evidence from China

**DOI:** 10.1186/s13561-026-00718-y

**Published:** 2026-01-20

**Authors:** Di Yang, Yubraj Acharya

**Affiliations:** 1 LSE Health, London School of Economics and Political Science, Houghton Street, London, WC2A 2AE UK; 2 Department of Health Policy and Administration, The Pennsylvania State University, 601L Ford Building, University Park, PA 16801 USA

**Keywords:** Housing demolition, Development-induced displacement, Medical utilization, Medical expenditure, Self-rated health, Mental health

## Abstract

**Background:**

China’s fast economic growth has been accompanied by rapid urbanization and urban renewal. Millions of households have experienced housing demolition and relocation (“chaiqian”) to vacate the land for urban renewal and infrastructure projects. Housing demolition can be a major life disruption and place a considerable burden on both mental and physical health. Meanwhile, replacement housing, provided as compensation for demolition, can improve housing quality and access to care, thus improving health.

**Methods:**

Using data from the China Family Panel Studies and an event study model with a staggered difference-in-differences framework, we examined the effects of housing demolition on individuals’ medical utilization in the year of demolition, as well as two and four years afterward. Medical utilization was measured as whether an individual uses medical services (incurring medical expenditure) and the amount of medical expenditure if using medical services. We also explored the effects of housing demolition on health measures, namely self-rated health and mental health status, as potential mechanisms through which housing demolition affects medical utilization.

**Results:**

Overall, housing demolition did not affect whether an individual used medical services. However, conditional on using medical services, housing demolition increased the amount of medical expenditure by approximately 1,639 CNY (234 USD) two years after demolition. We did not find evidence that housing demolition is associated with self-rated health or mental health status. Moreover, we found urban-rural heterogeneity in the effects of housing demolition. Rural residents have a higher likelihood of using medical services and higher medical expenditure two years after demolition, while urban residents have a lower likelihood of using medical services four years after demolition.

**Conclusions:**

Our findings highlight the importance of housing as a social determinant of health and contribute to the growing literature on development-induced displacement. The increased medical expenditure after housing demolition calls for a multidimensional evaluation of compensation for housing demolition. The compensation should consider both the loss of property itself and other associated adverse impacts, such as on health and medical care, to fully offset the burden of housing demolition, especially for rural residents who are particularly vulnerable after housing demolition.

## Introduction

China’s fast economic growth has been accompanied by rapid urbanization and urban renewal. As a result, millions of households have experienced “chaiqian”, which is housing demolition and relocation to vacate the land for infrastructure projects and urban renewal [1]. Although the affected households usually receive replacement housing or monetary compensation, they may face various challenges, such as the loss of social connections and the increased allostatic load associated with resettlement and possible migration.

Several studies, in both Chinese and international contexts, have examined the impact of housing demolition and relocation (sometimes termed “development-induced displacement” in the international context) on various outcomes, primarily labor supply. Among the studies that attempt to establish causality, Picarelli (2019) uses a fuzzy regression discontinuity design to estimate the impact of a housing relocation program in South Africa. The study uses program eligibility, the income threshold, as the cutoff for discontinuity and finds a reduction in labor supply among the households affected by housing demolition [2]. In China, Li et al. (2019) use a difference-in-differences model to estimate the effect of housing demolition on labor supply. They find a significant growth in household wealth as a result of the compensation provided for housing demolition, in the form of replacement housing and/or monetary compensation. This wealth, in turn, reduces the individual’s labor supply and increases life satisfaction among the affected households [3]. Similarly, Zhao & Liu (2022) use a difference-in-differences model to examine the effect of housing demolition in China [4]. They also find that housing demolition reduces labor participation among the affected households.

Outside of China, a few studies have examined the influence of neighborhood renewal and found mixed effects on health outcomes [5, 6]. Blackman et al. (2001) find that neighborhood renewal in Newcastle, UK, is associated with improved mental health but not with respiratory health or health care utilization. Similarly, Egan et al. (2013) show that urban neighborhood demolition and housing improvement programs in Glasgow, UK, do not affect physical health and have a positive effect on mental health. Moreover, a growing body of development economics literature has examined the effects of development-induced resettlement, yet a consensus on its impacts remains elusive [7, 8]. Mayer et al. (2023) find that individuals affected by housing demolition and resettlement due to large hydropower projects in Brazil have worse mental health outcomes despite receiving compensation. In contrast, Randell (2016) conducted a longitudinal survey of households affected by the construction of Belo Monte Dam in Brazil and found that subjective well-being improved for most households. Our study contributes to this ongoing debate on the impacts of development-induced displacement.

A few studies have examined the effects of housing demolition on other outcomes, such as life satisfaction, consumption, investment behavior, and inequality, in both Chinese and international contexts [9–12]. Among these studies, Hu et al. (2022) find no significant association between housing demolition and life satisfaction in China. They attribute the null results to two counteracting effects of housing demolition: On the one hand, housing demolition and the resulting shift to new, higher-quality housing can raise life satisfaction. On the other hand, housing demolition and relocation can reduce job stability and disrupt social connections, thus reducing life satisfaction. Yuan & Huang (2018) analyze data from the China Household Finance Survey and find that housing demolition can significantly increase the weight of risky assets (for example, stocks) in the financial investment portfolio. They argue that this effect can be explained by the “mental accounting” theory. Specifically, compared to their regular income, households may be less risk-averse when investing the unexpected monetary compensation. This finding is consistent with that of Yuan et al. (2021), who find that housing demolition increases conspicuous consumption. Similarly, Chai (2014) examines the effect of housing demolition on consumption and the moderating effect of household assets. The findings suggest that, after housing demolition, households with multiple houses beforehand increase their consumption level significantly, but those with only one house decrease consumption, because the latter lose their only house and need to rent another place [12].

In this study, we used three waves of survey data from the China Family Panel Studies (CFPS) and examined the overall effect of housing demolition on medical utilization. For auxiliary analysis, we explored the association between housing demolition and health measures, namely self-rated health and mental health status, as potential intermediary mechanisms for how housing demolition affects medical utilization. We used an event study model with a staggered difference-in-differences framework. To preview the results, housing demolition does not affect whether an individual uses medical services. However, conditional on using medical services (incurring a non-zero medical expenditure), housing demolition increases the amount of medical expenditure two years after demolition. Our results also show that affected individuals in rural areas are more vulnerable than those in urban areas, as they experience both an increased likelihood of using medical services and a higher amount of medical expenditure (again, conditional on incurring an expenditure).

Our study makes three key contributions to the literature, in addition to contributing to the ongoing debate on the impacts of development-induced displacement. First, we examine the effect of housing demolition on medical utilization and explore the potential mechanism through health outcomes. Medical utilization and health outcomes heretofore have been under-researched in the literature on housing demolition, despite the centrality of these outcomes in affecting downstream measures, such as labor force participation, which other studies have examined. Second, we leverage an event study model with a staggered difference-in-differences framework to investigate the effects of housing demolition at multiple time points: in the year of demolition, two years after demolition, and four years after demolition, thus offering insights into the temporal aspect of the effects (see Methods). Finally, our study contributes to the emerging literature on the multidimensional evaluation of compensation for housing demolition [13]. Our findings imply that the amount of compensation should not solely depend on the size or market value of the demolished property. Policymakers should also consider other potential adverse effects of housing demolition, such as on health and medical utilization. Moreover, our findings have broader implications for other LMICs currently under rapid urbanization, industrialization, and economic transformation, such as Vietnam.

### Institutional background of housing demolition and relocation (“Chaiqian”)

In China, a government body at the county level or above has the power to expropriate private real estate built on the state-owned land with fair compensation for what is called “public interest” [14]. Typical examples of public interest include building new infrastructure projects, such as airports, highways, and bridges [15]. In practice, public interest also includes improving community housing conditions, as in urban renewal programs, such as the nationwide Shantytown Redevelopment Program [16]. A shantytown (“chengzhongcun”, which literally translates to “village within city”) is a high-density living community with inadequate infrastructure (including medical facilities) and poor sanitation [17]. The Shantytown Redevelopment Program would demolish the shantytown and build new apartments and community amenities, such as a community clinic, in the area.

Irrespective of the reason for housing demolition, the compensation is usually replacement housing, a lump sum of money, or a combination of both [4, 18, 19]. The amount of compensation can be significant. Using the 2011 China Household Finance Survey data, Hu et al. (2022) report that the average compensation was 179,600 CNY (1 USD = 7 CNY), more than eight times the average disposable income per capita that year (21,809 CNY). Nevertheless, not all households are willing to accept the appropriation of their property. Compulsory demolition would spark social conflict and cause mental distress on the affected household members [20, 21]. Although different bottom-up institutional arrangements for Shantytown Redevelopment have emerged in recent years, the top-down approach remains dominant in China, despite high transaction costs [22].

Housing demolition and relocation have affected millions of households in China. The Shantytown Redevelopment Program alone initiated the construction of 1.65 million housing units in 2021 [1]. The 2010 baseline survey of the China Family Panel Study shows that approximately 6.6% of the surveyed households had ever experienced housing demolition and relocation [23].

## Methods

### Data

We use data from the China Family Panel Studies, which is a nationally representative, biannual, and longitudinal survey. The survey has been tracking the same set of households since 2010 and includes questions on health, housing, socioeconomic background, and demographic characteristics [24]. The dataset can be accessed with approval from the Institute of Social Science Survey at Peking University. We use the 2014, 2016, and 2018 datasets for the analysis because the 2012 survey does not include questions on housing demolition. The 2010 survey asks whether the household has *ever* experienced housing demolition, instead of housing demolition experience in that year, which precludes its use for examining the same-year effect of housing demolition.

We use a full sample of 19,458 individuals for the main analysis and a balanced panel of 10,360 individuals for the robustness check. We will address the issue of high attrition in the Discussion section. Table 1 reports the 2018 characteristics of the full sample as well as individuals who reported housing demolition in 2018 (193 individuals). In the full sample, 72% of the individuals incur any medical expenditures, and the average annual total medical expenditure is 3,692 CNY. Approximately 65% of the sample reports a health status of three or higher on a scale of one (“poor”) to five (“excellent”). Hereafter, self-rated health of three or higher is referred to as good health (three is “good” on the scale, which is also the median and most common response of the sample). About 49% of the sample are male, 90% are married, and the average age is 52 years. The average educational attainment is 7 years. The average household income is CNY 88,230. Approximately one-sixth of the sample consumes alcohol more than three times a week, and 31% have a history of smoking. About 7% of the sample is uninsured; 74% of the sample is insured with the New Cooperative Medical Scheme, and the rest is insured with the Urban Resident or Employee Basic Medical Insurance. About 3% of the sample has experienced housing demolition during the surveyed period (2014–2018), as housing demolition is a lower-probability event.

**Table 1:** Comparison of 2018 characteristics between individuals who experienced housing demolition in 2018 and all individuals

Characteristics	Experienced Demolition	All Individuals	*p*-value
Use medical services (incur medical expenditure, binary)	68.4%	72.0%	0.27
Total annual medical expenditure (CNY), conditional on incurring non-zero medical expenditure	4003.59 (13923.77)	3691.77 (12953.28)	0.74
Self-rated health			
1 (worst)	18.1%	20.0%	0.23
2	20.7%	15.1%	
3	39.4%	40.1%	
4	9.8%	12.8%	
5 (best)	11.9%	12.0%	
Sex (male)	49.7%	48.8%	0.80
Age, years	52.93 (14.25)	52.38 (13.66)	0.58
Marital status			
Never married	2.6%	3.9%	0.06
Married	88.1%	89.5%	
Cohabit	1.0%	0.2%	
Divorced	2.6%	1.2%	
Widowed	5.7%	5.1%	
Years of education	7.64 (4.81)	7.03 (4.70)	0.07
Annual household income (1,000 CNY)	440.75 (1022.38)	88.23 (162.74)	< 0.001***
Compensation for housing demolition (1,000 CNY)	403.2 (1052.1)	NA	
Alcohol consumption (drink more than 3 times a week in the past month)	18.7%	16.7%	0.46
Had a history of smoking	36.3%	31.2%	0.13
Social health insurance type			
Uninsured	6.7%	6.6%	< 0.001***
Urban Resident Basic Medical Insurance	15.0%	7.0%	
Urban Employment Basic Medical Insurance	16.6%	12.4%	
New Cooperative Medical Scheme	61.7%	74.0%	
GDP per capita (CNY)	58168.25 (28850.75)	58548.10 (26966.46)	0.85
N	193	12,676	

Notes: “Experienced demolition” refers to the individual who report demolition in 2018. “All individuals” refers to all individuals in the sample. Standard deviations are reported in brackets. 1 USD = 7 CNY. The p-values reported in the final column are from a formal test (t-test for continuous and chi-squared test for categorical variables) between individuals who experienced demolition and the overall sample. * *p* < 0.05, ** *p* < 0.01, *** *p* < 0.001

Table 1 further shows that individuals affected by housing demolition in 2018 do not differ significantly from the overall population in most characteristics, except for the type of social health insurance and household income. The significant difference in social health insurance type likely reflects the difference in area of residence (rural or urban), making the heterogeneity analysis by urban versus rural status appropriate. The higher household income among those who experienced a demolition can be explained by the compensation they received. The individuals who experienced demolition in 2018 report an average compensation of 403,000 CNY (Valuation for replacement housing is self-reported. 1 USD = 7 CNY).

We also compare the 2014 characteristics of individuals who were never affected by housing demolition (never-demolished) and individuals who reported housing demolition in 2016 or 2018 (to-be-demolished) in Appendix Table 1. The results indicate that individuals who subsequently experienced housing demolition do not differ significantly from those who did not experience housing demolition in key characteristics, such as household income. The only significant difference is in the type of social health insurance, which reflects China’s three-tier insurance programs (before the consolidation of two of them in 2016) and the higher propensity of the urban population to face housing demolition because of urban renewal—further supporting the rationale for examining heterogeneous effects by urban versus rural status.

### Theoretical framework

**Fig. 1:**
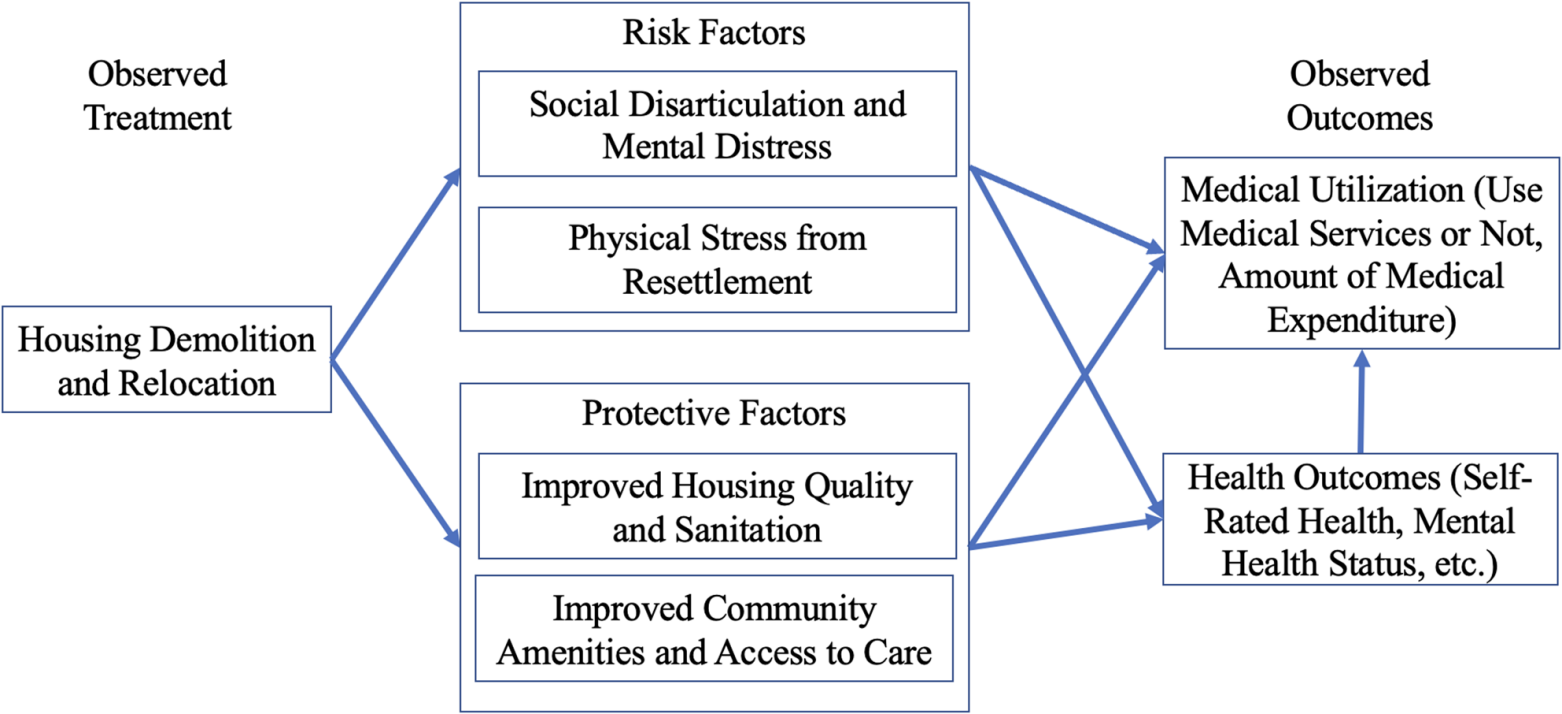
Theoretical Framework

In order to elucidate the risk factors and protective factors through which housing demolition can influence health and medical utilization, in Fig. 1, we present a modified version of the impoverishment risks and reconstruction (IRR) model [25]. Housing demolition can uproot households from their social community and weaken the social support available to them [26]. The effect of a broken social network may be particularly pronounced for individuals who have no prior intention to move [27]. Moreover, literature shows that rural residents often migrate to urban areas after housing demolition [28]. The physical and mental stress associated with migration and resettlement would increase the allostatic load on the affected individuals. These factors can have a negative impact on health. Conversely, with better replacement housing and sanitation conditions, individuals can be better off than before, particularly in urban renewal programs, such as Shantytown Redevelopment [29].

*Ex ante*, it is difficult to predict the net effect of these mechanisms. However, some hypotheses can be made on how the factors may play out differently for urban and rural residents and on the potential temporal dimension of the effects. The protective factors above are likely stronger for urban renewal programs in urban areas. Therefore, rural residents are likely more vulnerable than urban residents after housing demolition. Temporally, for rural residents, it is possible that they face negative effects in the short term as they move to and navigate their new place. For urban residents, conversely, the negative effects may be outweighed by the protective factors mentioned above—leading to net positive effects, at least in the longer term.

### Empirical strategy

We used an event study model with a staggered difference-in-differences framework to account for individuals experiencing housing demolition in different years [30]. The individuals who have never experienced housing demolition are the control group. The individuals who experienced housing demolition in any year of the studied period (2014–2018) are the treatment group.

We analyzed three outcomes: medical utilization for the main analysis, and self-rated health and mental health as potential mechanisms. Medical utilization was measured annually as whether an individual uses medical services (incurring medical expenditure) and the amount of medical expenditure if using medical services. Total annual medical expenditure is readily available in the dataset and is the sum of medical expenditure from hospitalization and outpatient costs.

Our primary independent variables are a series of dummy variables indicating housing demolition experience. In the survey, respondents were asked, “In the past 12 months, did your household experience housing demolition/relocation?” Following the event study methodology, we created a series of dummy variables that indicate the time relative to housing demolition.

The regression equation for the event study model takes the following form:

$$\begin{aligned}\:{Y}_{it}&=\alpha\:+\:{{\beta\:}_{1}Lead4Years}_{it}+{{\beta\:}_{2}EventYear}_{it}+{{\beta\:}_{3}Lag2Years}_{it}\\&+{{\beta\:}_{4}Lag4Years}_{it}+{{\beta\:}_{x}X}_{it}+{\lambda\:}_{i}+{\mu\:}_{t}+{\epsilon\:}_{it}\end{aligned}$$

In this equation, *Y*_*it*_ is the outcome variable for individual *i* in survey year *t*. *Lead4Years*_*it*_, *EventYear*_*it*_, *Lag2Years*_*it*_, and *Lag4Years*_*it*_ are a set of binary variables indicating demolition experience for individual *i* in survey years *t*. For example, *Lead4Years*_*it*_ takes the value of 1 if individual *i* experienced housing demolition in survey year *t + 4* (four-year lead), and 0 otherwise. Likewise, *Lag2Years*_*it*_ takes the value of 1 if individual *i* experienced housing demolition in survey year *t-2* (two-year lag), and 0 otherwise. *λ*_*i*_ and $\:{\mu\:}_{t}$ are individual fixed effects and time fixed effects. *X*_*it*_ includes time-varying individual characteristics (demographic and health behavior-related) that can affect the outcomes, including age, marital status, years of education, annual household income, alcohol consumption, smoking history, and the type of social health insurance. We also control for GDP per capita at the provincial level, as the dataset does not provide identifiable geographic information below the provincial level. *ε*_*it*_ is the error term.

In the regression equation, *β*_*2*_, *β*_*3*_, and *β*_*4*_ are the key coefficients of interest and represent the plausibly causal effect of housing demolition on the outcomes in the year of demolition, 2 years after demolition, and 4 years after demolition, respectively. To interpret *β*_*1*_, *β*_*2*_, and *β*_*3*_ as the plausibly causal effect, we need to assume that, in the absence of housing demolition, an individual would have had the same outcomes across the three survey rounds. Moreover, the health effect of housing demolition follows the same temporal trajectory, regardless of the year in which demolition occurs. This second assumption is due to the “staggered exposure”, i.e., that housing demolition can occur in 2014, 2016, or 2018 [31]. *β*_*1*_ helps us gauge the parallel trend assumption (see Robustness Check section).

Following existing literature on modeling variables with a mass at zero, we use a two-part model to analyze medical utilization [32–36]. The first part models whether an individual used medical services. The second part models the amount of medical expenditure conditional on using medical services (i.e., having a positive medical expenditure). For better interpretability, we first used a linear probability model to analyze whether an individual used medical services and, therefore, incurred medical expenditure. Then, for the second part, again following the existing literature, we took the natural logarithm of total annual medical expenditure as the dependent variable and used OLS. To clarify, the use of a two-part model obviates the need to take the natural logarithm of zero (which in turn would lead to a loss of a large number of observations) or to arbitrarily assign a small positive value before taking the logarithm.

## Results

### Main results

Table 2 reports coefficients and standard errors from the main analysis. Column 1 shows the results from the first part of the model, i.e., for “whether individuals used medical services” (incurred medical expenditure) as the outcome. Column 2 shows the results from the second part, i.e., answering the question of *how much* medical expenditure they incurred, conditional on incurring a non-zero medical expenditure. Note that in Column 2, the sample size is smaller because it does not include individuals who did not use medical services. In Column 1, we do not observe a significant impact on whether the affected individual used medical services at any time point after housing demolition. However, conditional on using medical services, the *amount* of expenditure two years after housing demolition is positively associated with housing demolition (Column 2). In terms of the magnitudes, individuals who experienced housing demolition two years earlier incur 44.4% more (approximately 1,639 CNY) annual medical expenditure than those who did not. This represents about 2% of the average household income, as medical expenditure constitutes only about 6% of household income in China [37].

**Table 2:** Effect of housing demolition on medical utilization (event study model)

	Whether using medical services (binary)	Log(total annual medical expenditure)
Four years before housing demolition	0.010	0.134
	(0.038)	(0.180)
Two years before housing demolition (reference)		
Year of housing demolition	−0.013	0.094
	(0.030)	(0.140)
Two years after housing demolition	0.032	0.444*
	(0.038)	(0.172)
Four years after housing demolition	−0.083	−0.133
	(0.055)	(0.223)
Demographic covariates	controlled for	controlled for
Health-related covariates	controlled for	controlled for
Sample size	44,752	31,793

Notes: Standard errors are clustered at the county level and reported in brackets. The survey is conducted every two years, so the interval in this table is two years. Column 2 regression was conducted on a subsample of individuals who used medical services. Demographic covariates include age, years of education, marital status, and log(household income). Health-related covariates include alcohol consumption, smoking history, and the type of social health insurance. These covariates correspond to X*it* in the regression equation. *p* < 0.05, ** *p* < 0.01, *** *p* < 0.001

### Robustness checks

**Fig. 2:**
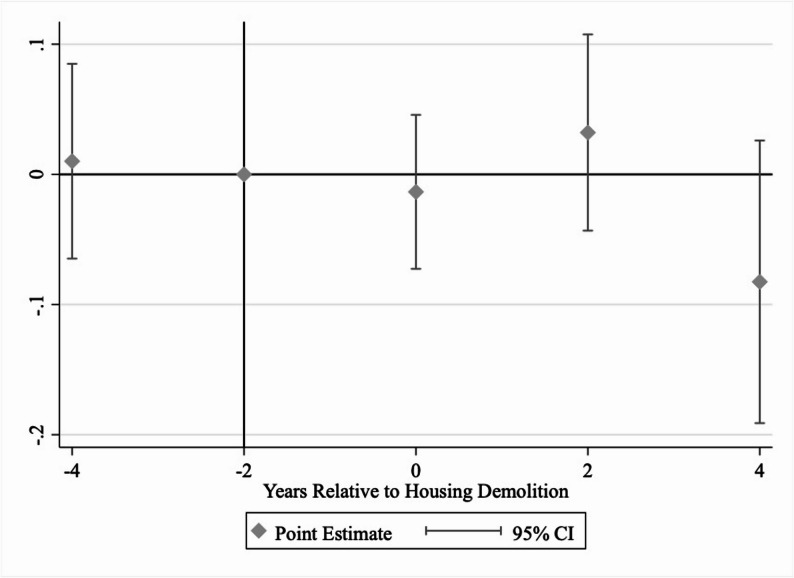
Likelihood of Using Medical Services (Binary) Before and After Home Demolition Notes: This figure shows the difference in the likelihood of using medical services (incurring medical expenditure) between those who experienced housing demolition in any year of the survey and those who did not. The reference period is two years before housing demolition. The survey was conducted every two years, hence the two-year intervals in the figure

**Fig. 3:**
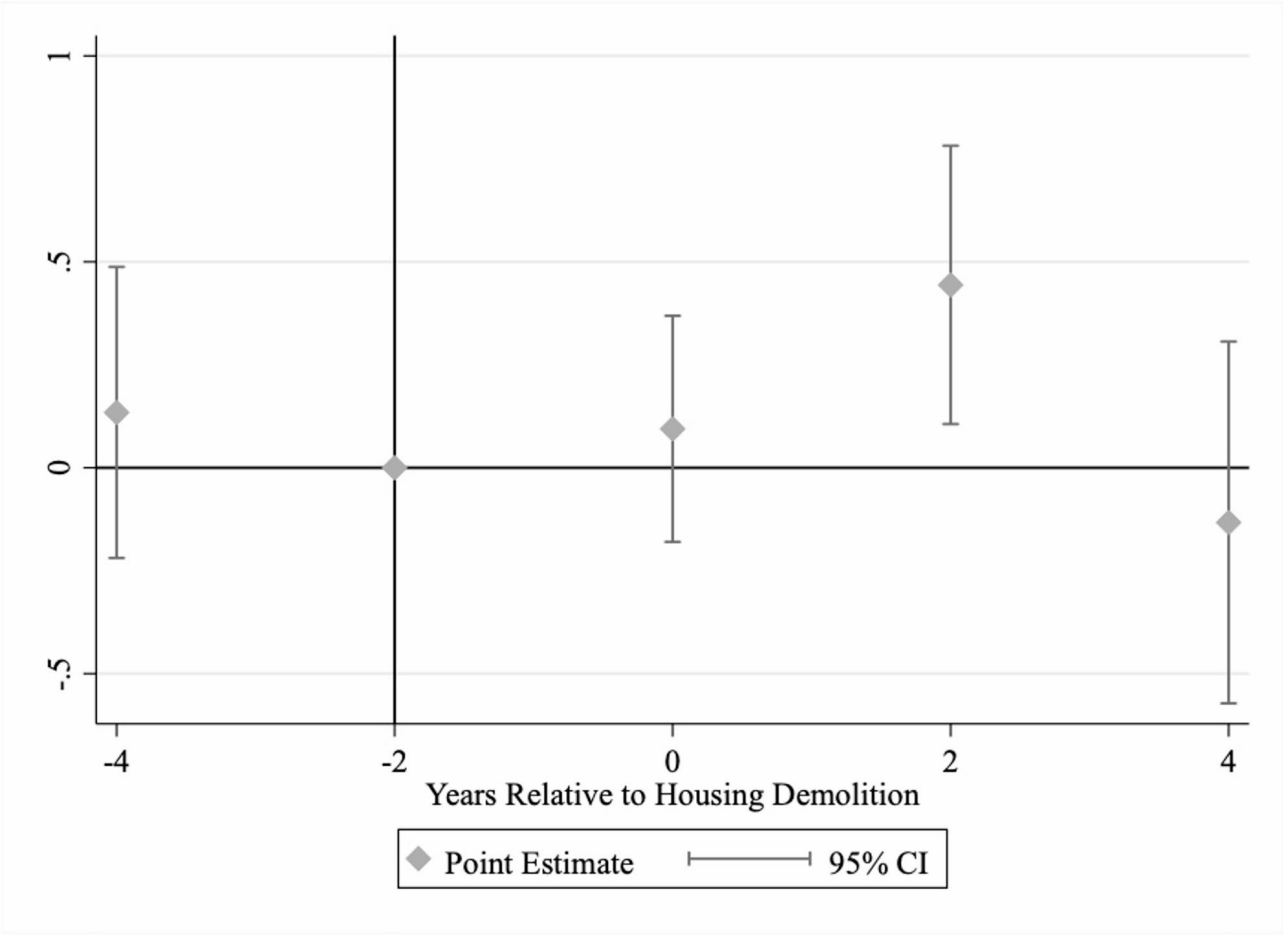
The Amount of Medical Expenditure Before and After Home Demolition, Conditional On Incurring Non-Zero Medical Expenditure Notes: This figure shows the difference in the medical expenditure (logarithm-transformed) between those who experienced housing demolition in any year of the survey and those who did not. The reference period is two years before housing demolition. The survey was conducted every two years, hence the two-year intervals in the figure

A key assumption for the difference-in-differences framework is the parallel trend (i.e., whether *β*_*1*_ in the regression equation is equal to zero). To test this assumption, we plotted the estimates and 95% confidence intervals from the event study model, using two years before housing demolition as the baseline (Figs. 2 and 3). Figure 2 shows that the difference in the likelihood of using medical services between those who experienced demolition and those who did not change significantly from four years before housing demolition to two years before demolition, which suggests that the parallel trend assumption is not violated. Similarly, Fig. 3 shows that the difference in the amount of medical expenditure between the two groups did not change significantly from four years before housing demolition to two years before demolition, which again suggests no violation of the parallel trend assumption. Consistent with the graphs, coefficients for $\:{Lead4Years}_{it}$ in Table 2 are statistically not significant.

As another robustness check for the methodology, we also used an individual fixed effects model to analyze the effects of housing demolition on whether using medical services and the amount of medical expenditure, conditional on using medical services. In this analysis, the effect of housing demolition is estimated using within-individual variation in home demolition and the outcomes. The results, reported in Appendix Table 2, are consistent with the results from the event study model.

A major concern with analysis involving panel data is potential differential attrition. Differential attrition based on outcome variables would bias our estimates. For example, if those with higher medical expenditure were more likely to remain in the sample across survey years and also more likely to experience home demolition, our estimates might be biased upward. To assess the extent of differential attrition, we ran a regression of a binary measure of attrition (coded as 0 if the individual has data available for all years of survey, 1 if the individual has data available in 2014 but missing in 2016, 2018, or both 2016 and 2018) on self-rated health, medical expenditure, and interacted these two variables with housing demolition. We report the results in Appendix Table 3, which shows that individuals who experienced housing demolition are indeed more likely to drop out of the survey than those who did not. However, the non-significant coefficients of the interaction terms suggest no differential attrition based on outcome variables between individuals who experienced housing demolition and those who did not. Therefore, attrition is unlikely to bias our findings significantly.

To further alleviate the attrition concern, we run the same set of regressions as in the main analysis but with the balanced sample. Findings from the balanced sample analysis are similar to those from the main analysis using the full sample (Appendix Table 4). Housing demolition is not associated with the likelihood of incurring medical expenditure or self-rated health, but increases the amount of medical expenditure two years after demolition.

### Exploratory analysis of the mechanism

One possible mechanism for the increased medical expenditure is that the physical and mental distress from the housing demolition and relocation process can cause deterioration in health, which in turn increases medical utilization. In this section, we report results from an exploratory analysis on the relationship between housing demotion and physical and mental health.

Self-rated health is often used as a key indicator of health status. In the CFPS survey, respondents were asked how they would rate their health status on a scale of one to five (five being the best). We followed the common approach in the literature and recoded self-rated health into a binary variable such that a response of three and above counted as good health [38]. We use three as the cutoff, as it is the median and the most common response. We run the same regression as specified in the main analysis and report the results on self-reported health in Table 3. The results for the urban subsample suggest that housing demolition does not have a significant impact on self-rated health (column 2). The results for the overall sample (column 1) and rural subsample (column 3) are inconclusive because the parallel trend assumption is violated, as indicated by the statistically significant coefficients for four years before housing demolition ($\:{Lead4Years}_{it})$.

**Table 3:** Effect of housing demolition on self-rated health

	Overall	Urban	Rural
Four years before housing demolition	−0.079*	−0.048	−0.116*
	(0.034)	(0.053)	(0.047)
Two years before housing demolition (reference)			
Year of housing demolition	−0.038	−0.008	−0.086
	(0.027)	(0.036)	(0.052)
Two years after housing demolition	−0.046	−0.003	−0.122
	(0.039)	(0.039)	(0.082)
Four years after housing demolition	−0.016	0.027	−0.073
	(0.056)	(0.062)	(0.122)
Demographic covariates	controlled for	controlled for	controlled for
Health-related covariates	controlled for	controlled for	controlled for
Sample size	44,752	19,219	25,533

Notes: Standard errors are clustered at the county level and reported in brackets. The survey is conducted every two years, so the interval in this table is two years. Demographic covariates include age, years of education, marital status, and log(household income). Health-related covariates include alcohol consumption, smoking history, and the type of social health insurance. These covariates correspond to X*it* in the regression equation. Self-rated health is a binary variable, coded 1 if the respondent rates his/her health as “good” or higher. * *p* < 0.05, ** *p* < 0.01, *** *p* < 0.001

Change in mental health is another key mechanism through which home demolition can alter medical expenses. However, only the 2014 survey included measures of mental health. Therefore, we were unable to conduct an analysis similar to the one for self-reported health. Nonetheless, for 2014, the survey included a six-question Kessler Psychological Distress Scale, where the respondents were asked how often they experienced certain feelings. In Table 4, we report the descriptive statistics of the answers by demolition experience and conduct chi-square tests to compare responses of those who experienced home demolition to those who did not. The p-values for chi-square tests suggest no significant difference in any of the mental health measures between individuals who experienced housing demolition and individuals who did not.

**Table 4:** Mental health assessment questions and score

Variables	Level	No Demolition	Demolition	*p*-value
How often during the past month did you feel depressed?	Almost every day	411 (2.4%)	8 (4.2%)	0.11
	Often	940 (5.5%)	12 (6.3%)	
	Half of the time	1086 (6.4%)	6 (3.2%)	
	Sometimes	6598 (38.8%)	82 (43.4%)	
	Never	7966 (46.9%)	81 (42.9%)	
How often during the past month did you feel nervous?	Almost every day	256 (1.5%)	5 (2.6%)	0.66
	Often	771 (4.5%)	7 (3.7%)	
	Half of the time	911 (5.4%)	9 (4.8%)	
	Sometimes	5309 (31.2%)	63 (33.3%)	
	Never	9767 (57.4%)	105 (55.6%)	
How often during the past month did you feel restless or fidgety?	Almost every day	287 (1.7%)	5 (2.6%)	0.85
	Often	642 (3.8%)	8 (4.2%)	
	Half of the time	817 (4.8%)	10 (5.3%)	
	Sometimes	4720 (27.8%)	53 (28.0%)	
	Never	10,543 (62.0%)	113 (59.8%)	
How often during the past month did you feel hopeless?	Almost every day	210 (1.2%)	4 (2.1%)	0.67
	Often	430 (2.5%)	3 (1.6%)	
	Half of the time	511 (3.0%)	5 (2.6%)	
	Sometimes	2648 (15.6%)	33 (17.5%)	
	Never	13,205 (77.7%)	144 (76.2%)	
How often during the past month did you feel that everything was an effort?	Almost every day	375 (2.2%)	4 (2.1%)	0.98
	Often	796 (4.7%)	8 (4.2%)	
	Half of the time	869 (5.1%)	11 (5.8%)	
	Sometimes	4623 (27.2%)	49 (25.9%)	
	Never	10,351 (60.8%)	117 (61.9%)	
How often during the past month did you feel that life was meaningless?	Almost every day	190 (1.1%)	2 (1.1%)	0.28
	Often	422 (2.5%)	0 (0.0%)	
	Half of the time	556 (3.3%)	5 (2.6%)	
	Sometimes	2518 (14.8%)	29 (15.3%)	
	Never	13,318 (78.3%)	153 (81.0%)	
Mental health score		20.78 (3.98)	20.68 (4.34)	0.73
Serious mental illness (score < 13)		893 (5.2%)	12 (6.3%)	0.50
N		17,028	189	

Notes: This table presents the six questions of Short Kessler Psychological Distress Scale (K6), the score calculated from the scale, and whether the score suggests serious mental illness. These variables are only available for 2014. The last column reports the p-values for the chi-square tests for each mental health assessment questions and p-values for the t-tests for mental health score and whether the score suggests serious mental illness

We followed the methodology of the Kessler Psychological Distress Scale and calculated the psychological distress score from this scale using 13 as the cutoff for serious mental illness [39]. We conducted t-tests for the overall distress score and whether the respondent is classified with serious mental illness. The results remain the same. Overall, an individual’s health status alone does not appear to be the primary mechanism through which housing demolition affects medical expenditure, although this claim comes with significant caveats.

### Urban-rural heterogeneity in the effects

Motivated by the theoretical framework and given the vast differences between urban and rural populations in China, we may expect the effect of housing demolition to differ between these two populations. In fact, as reported in Appendix Table 1, the distribution of social health insurance types—which in China’s context also reflects whether one lives in an urban or a rural area—in 2014 differs significantly between individuals who subsequently experienced housing demolition and those who did not.

Table 5 reports the effect of housing demolition on the outcomes, separately for urban (Panel A) and rural (Panel B) subsamples. For the urban subsample, the protective factors of housing demolition and relocation (Fig. 1) seem to prevail, and individuals who experienced housing demolition have a 20.5% points lower likelihood of using medical services four years after demolition than those who did not. Housing demolition is not associated with the amount of medical expenditure. For the rural subsample, housing demolition is associated with both increased likelihood of using medical services and higher amount of total annual medical expenditure two years after housing demolition, which suggests rural residents are particularly vulnerable after housing demolition. Experiencing housing demolition increases the likelihood of incurring medical expenditure by 17% points in rural areas two years after demolition. Individuals who experienced housing demolition incur 64.7% points higher annual medical expenditure than those who did not.

**Table 5:** Effect of housing demolition on medical utilization (urban and rural subsamples)

	Whether use medical services (binary)	Log(total annual medical expenditure)
Panel A: urban subsample		
Four years before housing demolition	0.001	−0.084
	(0.070)	(0.302)
Two years before housing demolition (reference)		
Year of housing demolition	−0.002	0.281
	(0.050)	(0.178)
Two years after housing demolition	−0.033	0.370
	(0.054)	(0.222)
Four years after housing demolition	−0.205**	−0.163
	(0.077)	(0.301)
Demographic covariates	controlled for	controlled for
Health and habit related covariates	controlled for	controlled for
Sample size	19,219	13,301
Panel B: rural subsample		
Four years before housing demolition	0.024	0.245
	(0.038)	(0.249)
Two years before housing demolition (reference)		
Year of housing demolition	−0.012	0.012
	(0.044)	(0.224)
Two years after housing demolition	0.171*	0.647*
	(0.076)	(0.315)
Four years after housing demolition	0.104	0.135
	(0.083)	(0.377)
Demographic covariates	controlled for	controlled for
Health and habit related covariates	controlled for	controlled for
Sample size	25,533	18,492

Notes: Standard errors are clustered at the county level and reported in brackets. The survey is conducted every two years, so the interval in this table is two years. Column 2 regression was conducted on a subsample of individuals who used medical services. Demographic covariates include age, years of education, marital status, and log(household income). Health-related covariates include alcohol consumption, smoking history, and the type of social health insurance. These covariates correspond to X*it* in the regression equation. * *p* < 0.05, ** *p* < 0.01, *** *p* < 0.001

## Discussion

Our study contributes to the emerging literature on the multi-faceted effects of housing demolition in China and, more generally, development-induced displacement and resettlement in low- and middle-income countries. Only a few studies have investigated the effects of development-induced displacement on health measures, and the findings to date remain inconclusive [7, 8]. Our findings are consistent with the existing literature, which shows that urban renewal does not affect whether individuals use medical services per se [40]. However, in contrast to what the urban renewal literature argues, we did not observe improvement in mental health following housing demolition and relocation [40, 41]. Our research broadly suggests that the compensation for housing demolition should consider not only the value or size of the demolished property, but also other downstream effects, such as the potential rise in medical expenditure.

Among our primary results, we find that housing demolition increases the amount of medical expenditure two years after the demolition. The urban-rural heterogeneity analysis suggests this increase in medical expenditure was primarily driven by rural residents and was accompanied by an increase in the likelihood of using medical services. Our mechanism analyses show no significant correlation between housing demolition and health outcomes, such as self-rated health or mental health status. However, prior literature suggests that rural residents often migrate to urban areas after housing demolition [28]. This helps explain findings for the rural population. As medical resources are heavily concentrated in urban areas in China [42], it is possible that rural residents increase medical utilization for previously unmet medical demands after migrating to urban areas and getting better access to care. This hypothesis would also explain why the increase in medical utilization does not happen immediately, but two years after housing demolition, as the migration and resettlement process can take time. For this rural population, after addressing the unmet medical demands, medical utilization seems to return to the normal level, as reflected in the no change in medical expenses between the year of demolition and four years after. For the urban subsample, the likelihood of using medical services declines four years after housing demolition. It appears that the benefits of improved housing quality and sanitation—the protective factors in our theoretical framework—materialize a few years after demolition. These explanations remain hypotheses for future research to investigate with more fine-grained data on medical utilization patterns, which the existing datasets lack.

Our findings should be interpreted in light of several limitations, which also point to areas for further research on this topic. First, the attrition across survey years is high, and individuals who experience housing demolition are more likely to drop out of the survey. This is a natural limitation for survey data, as individuals who experience housing demolition are more difficult to track and follow up with. Nevertheless, our attrition analysis shows no differential attrition based on the health outcomes between the individuals who experienced housing demolition and those who did not. Therefore, attrition is unlikely to bias our findings significantly. Consistent findings from the balanced panel and the full sample are also reassuring. Second, about 2% of the full sample reports housing demolition each year (200–300 observations), resulting in a small treatment group and raising the possibility of spurious effects. This limitation is inherent to the studies on housing demolition [43], because only a very small percentage of the general population experiences housing demolition. Our main finding—that medical expenditure conditional on incurring some medical expenditure rises after two years of home demolition—is stable across the battery of robustness checks we perform. Third, the timespan of our research is relatively short. A parallel trend test over a longer period would further enhance our confidence in the comparability between the treatment and control groups. Finally, our dataset does not contain information on the amount of compensation households receive, nor does it include details on the quality of replacement housing. Therefore, we cannot differentiate the effects of compensation and replacement housing from the effects of housing demolition itself. Instead, our study estimates the overall effect of housing demolition and relocation (chaiqian), which includes both the demolition and other concurring events associated with demolition, such as receiving compensation and moving into replacement housing. Future studies would require more detailed data, such as the reason for demolition, actual relocation distance, quality of replacement housing, timing and amount of compensation, pre-demolition housing conditions, and medical utilization patterns, to fully uncover mechanisms and parse out the effects of multiple changes that individuals experience in housing demolition. Furthermore, future researchers conducting their own surveys should confine the control and treatment groups to the same area, such as two neighboring communities where one was demolished and the other was not, to enhance comparability of the control and treatment groups.

## Conclusion

Overall, we find that housing demolition does not affect whether individuals use medical services. However, conditional on using medical services, housing demolition increases the amount of medical expenditure two years after demolition. We do not find evidence that housing demolition is associated with self-rated health or mental health status. We also find that rural individuals who experience housing demolition are more likely to use medical services two years after the demolition. In contrast, urban individuals experience a decline in the likelihood of using medical services four years after housing demolition, highlighting the potential protective effects of better quality of replacement housing and sanitation.

Our findings support a re-evaluation of how compensation for housing demolition is calculated. Under the current practice, compensation is based on the market value and the size of the demolished property. Given the multiple ways in which housing demolition can affect families beyond the direct loss of property, we support a multidimensional evaluation of compensation for housing demolition that considers both the loss of housing itself and other associated adverse impacts, such as health and medical utilization. This is more imperative for the rural population, whose lives are more likely to be disrupted than the urban population.

## Data Availability

The datasets analyzed in this study are available from the website of China Family Panel Studies (http://www.isss.pku.edu.cn/cfps/en/index.htm).

## Appendix

**Table:** Appendix Table 1. Comparison of 2014 Characteristics by Housing Demolition Experience

Characteristics	No Demolition	Demolition in 2016 or 2018	*p*-value
Incurring medical expenditure (binary)	71.3%	72.0%	0.80
Total annual medical expenditure (CNY)	2194.46 (8123.71)	2459.10 (8030.60)	0.60
Self-rated health			
1 (worst)	16.8%	19.0%	0.76
2	15.2%	15.3%	
3	36.0%	34.0%	
4	18.9%	17.2%	
5 (best)	13.1%	14.6%	
Sex (male)	48.6%	50.0%	0.66
Age, years	48.99 (13.33)	49.65 (14.33)	0.43
Marital status			
Never married	5.2%	4.9%	0.43
Married	89.9%	91.0%	
Cohabit	0.2%	0.0%	
Divorced	1.0%	1.1%	
Widowed	3.6%	3.0%	
Years of education	6.78 (4.62)	7.11 (4.74)	0.24
Annual household income (1,000 CNY)	52.20 (85.22)	54.76 (48.64)	0.63
Alcohol (drank more than 3 times a week in the past month)	16.6%	17.9%	0.58
Had a history of smoking	30.1%	29.1%	0.73
Social health insurance type			
Uninsured	6.0%	6.7%	< 0.001***
Urban Resident Basic Medical Insurance	6.2%	13.1%	
Urban Employment Basic Medical Insurance	11.2%	14.6%	
New Cooperative Medical Scheme	76.5%	65.7%	
GDP per capita (CNY)	42762.73 (18219.25)	43714.43 (21495.05)	0.40
N	10,038	268	

Notes: “No demolition” means the respondent did not report demolition in any year of the survey data (2014–2018). 1 USD = 7 CNY. The p-values reported in the final column are from a formal test (t-test for continuous and chi-squared test for categorical variables) between individuals who experienced demolition and the overall sample. * *p* < 0.05, ** *p* < 0.01, *** *p* < 0.001

**Table:** Appendix Table 2: Effect of Housing Demolition on Medical Expenditure (Individual Fixed Effects Model)

	Whether use medical services (binary)	Log(total annual medical expenditure)
No reported housing demolition experience (reference)		
Reported Housing demolition in the current survey	−0.016	0.060
	(0.027)	(0.130)
Reported Housing demolition in survey two years ago	0.030	0.422*
	(0.039)	(0.170)
Reported Housing demolition in survey four years ago	−0.085	−0.160
	(0.054)	(0.220)
Demographic covariates	controlled for	controlled for
Health and habit related covariates	controlled for	controlled for
Sample size	44,752	31,793

Notes: Standard errors are clustered at the county level and reported in brackets. The survey is conducted every two years, so the interval in this table is two years. Column 2 regression was conducted on a subsample of individuals who used medical services. Demographic covariates include age, years of education, marital status, and log(household income). Health-related covariates include alcohol consumption, smoking history, and the type of social health insurance. *p* < 0.05, ** *p* < 0.01, *** *p* < 0.001

**Table:** Appendix Table 3. Attrition Analysis

	Attrition
Housing demolition	0.370***
	(0.128)
Self-rated health	
1 (worst, reference)	
2	−0.032*
	(0.013)
3	−0.037***
	(0.012)
4	−0.010
	(0.013)
5 (best)	−0.025
	(0.015)
Interaction term of housing demolition and self-rated health	
Housing demolition * (self-rated health = 1), reference	
Housing demolition * (self-rated health = 2)	−0.039
	(0.130)
Housing demolition * (self-rated health = 3)	−0.125
	(0.109)
Housing demolition * (self-rated health = 4)	−0.114
	(0.134)
Housing demolition * (self-rated health = 5)	−0.237
	(0.158)
Log(medical expenditure)	−0.003**
	(0.001)
Housing demolition * Log(medical expenditure)	0.010
	(0.012)
Sample size	17,099

Notes: This table shows results from a regression of attrition on housing demolition and key characteristics of the individual, namely medical expenditure and health-related measures. Attrition is a binary variable, coded as 0 if the individual has data available for all years of survey, 1 if the individual has data available in 2014 but missing in 2016, 2018, or both 2016 and 2018. * *p* < 0.05, ** *p* < 0.01, *** *p* < 0.001

**Table:** Appendix Table 4. Effect of Housing Demolition on Medical Utilization (Balanced Sample)

	Whether using medical services (binary)	Log(total annual medical expenditure)
Four years before housing demolition	−0.002	0.185
	(0.041)	(0.177)
Two years before housing demolition (reference)		
Year of housing demolition	−0.043	0.153
	(0.033)	(0.141)
Two years after housing demolition	0.015	0.597**
	(0.044)	(0.188)
Four years after housing demolition	−0.117	0.086
	(0.073)	(0.326)
Demographic covariates	controlled for	controlled for
Health-related covariates	controlled for	controlled for
Sample size	44,755	31,796

Notes: Standard errors are clustered at the county level and reported in brackets. The survey is conducted every two years, so the interval in this table is two years. Column 2 regression was conducted on a subsample of individuals who used medical services. Demographic covariates include age, years of education, marital status, and log(household income). Health-related covariates include alcohol consumption, smoking history, and the type of social health insurance. These covariates correspond to Xit in the regression equation, which are the time-varying individual characteristics that can affect the outcomes. Self-rated health is a binary variable, coded 1 if the respondent rates his/her health as “good” or higher. * *p* < 0.05, ** *p* < 0.01, *** *p* < 0.001
